# RRW: repeated random walks on genome-scale protein networks for local cluster discovery

**DOI:** 10.1186/1471-2105-10-283

**Published:** 2009-09-09

**Authors:** Kathy Macropol, Tolga Can, Ambuj K Singh

**Affiliations:** 1 Department of Computer Science, University of California, Santa Barbara, CA 93106, USA; 2 Department of Computer Engineering, Middle East Technical University, 06531 Ankara, Turkey

## Abstract

**Background:**

We propose an efficient and biologically sensitive algorithm based on repeated random walks (RRW) for discovering functional modules, e.g., complexes and pathways, within large-scale protein networks. Compared to existing cluster identification techniques, RRW implicitly makes use of network topology, edge weights, and long range interactions between proteins.

**Results:**

We apply the proposed technique on a functional network of yeast genes and accurately identify statistically significant clusters of proteins. We validate the biological significance of the results using known complexes in the MIPS complex catalogue database and well-characterized biological processes. We find that 90% of the created clusters have the majority of their catalogued proteins belonging to the same MIPS complex, and about 80% have the majority of their proteins involved in the same biological process. We compare our method to various other clustering techniques, such as the Markov Clustering Algorithm (MCL), and find a significant improvement in the RRW clusters' precision and accuracy values.

**Conclusion:**

RRW, which is a technique that exploits the topology of the network, is more precise and robust in finding local clusters. In addition, it has the added flexibility of being able to find multi-functional proteins by allowing overlapping clusters.

## Background

In recent years, much effort has gone into finding the complete set of interacting proteins in an organism [1]. Such genome-scale protein networks have been realized with the help of high throughput methods, like yeast-two-hybrid (Y2H) [2,3] and affinity purification with mass spectrometry (APMS) [4,5]. In addition, information integration techniques that utilize indirect genomic evidence have provided both increased genome coverage by predicting new interactions and more accurate associations with multiple supporting evidence [6-9].

Complementary to the availability of genome-scale protein networks, various graph analysis techniques have been proposed to mine these networks for pathway or molecular complex discovery [10-15], function assignment [16-18], and complex membership prediction [19,20]. Bader and Hogue [21] propose a clustering algorithm to detect densely connected regions in a protein interaction network for discovering new molecular complexes. Spirin and Mirny [22] use superparamagnetic clustering (SPC) and a Monte Carlo (MC) algorithm to cluster a given protein interaction network. These algorithms work on undirected unweighted graphs and partition the network of proteins into non-overlapping clusters. However, genome-wide networks constructed with multiple supporting evidence have edges with varying degrees of confidence. The strength of confidence should be considered when identifying strongly connected proteins. Also, it is known that there are many multi-functional proteins which may play important roles in different functional modules. Therefore, a biologically more sensitive cluster identification technique should report clusters that may sometimes overlap. Several clustering techniques have since been proposed that take into account the given edge confidence [23] or overlapping clusters [24,25]. However, these algorithms all account for the two problems separately, and do not both use given biological edge confidences and find overlapping clusters at the same time.

In this paper, we propose a novel algorithm, repeated random walk (RRW for short), for molecular complex and functional module discovery within genome-scale protein interaction networks. This new algorithm utilizes both given edge weights and can find overlapping clusters. The idea is based on expansion of a given cluster to include the protein with the highest proximity to that cluster. Starting with a cluster of size one (any protein in the network), this iterative process is repeated either *k *times, or until a stopping condition is met, to obtain clusters of size ≤ *k*. All significant overlapping clusters are recorded and post-processed to remove redundant clusters based on a given overlap threshold. We use random walks with restarts to find the closest proteins to a given cluster. To increase the algorithm's speed, the random walk results from a given cluster are computed using linear combinations of precomputed random walk results obtained starting from single proteins. Unlike other techniques proposed for pathway discovery, the random walk method implicitly exploits the global structure of a network by simulating the behavior of a random walker [26].

We apply RRW on a genome-scale functional network of yeast genes and accurately identify statistically significant clusters of proteins. We validate the biological significance of the results by comparison to known complexes in the MIPS complex catalogue database [27]. By comparison to an existing clustering technique, we show that using edge weights in addition to connectivity information and allowing certain amounts of overlap between clusters are the key characteristics of RRW for finding biologically more significant clusters.

## Results and discussion

### Problem statement and algorithm

Let *G *= (*V*, *E*) be the graph representing a genome scale protein interaction network, where *V *is the set of nodes (proteins), and *E *is the set of *weighted *undirected edges between pairs of proteins. The edges are weighted by the strength of supporting evidence for functional association.

#### Problem definition

Given a physical protein interaction or predicted functional network of an organism, our goal is to find biologically significant groups of proteins in the network. Here, the definition of a biologically significant group entails proteins that function together in a biological pathway or are members of a protein complex. Moreover, significant clusters may contain proteins from different complexes, therefore revealing modular interactions at a higher level.

The problem can be stated formally as follows: Given an undirected weighted graph *G *= (*V*, *E*), find *top-m *connected clusters of vertices of size at most *k *where the ranking is based on statistical significance. (Assessment of statistical significance is discussed in detail at the end of this section.) Evaluating all possible sets of proteins for biological significance is obviously intractable, *O*(2^|*V*|^). Therefore, we propose a heuristic based on random walks on graphs. The idea is based on expansion of a given cluster to include the protein with the highest proximity to that cluster. Starting with a cluster of size one, this iterative process is repeated either *k *times, or until the next closest protein's distance is not within a given cutoff. In this way, clusters of size ≤ *k *are obtained (all intermediate clusters are also assessed for biological significance). Table 1 contains a reference of the various notations and symbols used throughout the paper.

**Table 1:** List of notations used

**Symbol**	**Definition**	**Symbol**	**Definition**
**G**	Undirected, weighted graph	*α*	Random walk restart probability

**V**	Vertices in graph	*λ*	Early cutoff value

**E**	Edges in graph	*k*	Number of iteractions (maximum cluster size)

**P**	Transition matrix for graph	**s_i_**	Restart vector for a node (or set of nodes) *i*

**C**	Vector consisting of a cluster of nodes	**x_i_**	Random Walk stationary vector from a node (or set of nodes) *i*

#### Random walks with restarts

**Figure 1:**
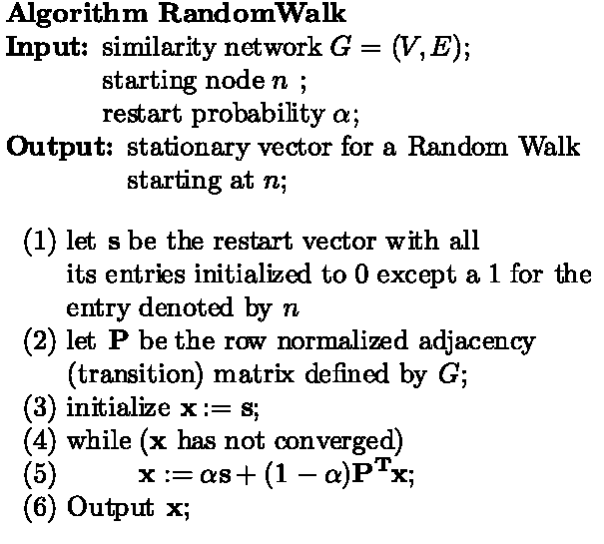
**Random walk algorithm**. Pseudocode for a random walk with restarts from a single vertex.

We use random walks with restarts for finding the highest affinity protein to a given cluster. The random walk technique exploits the global structure of a network by simulating the behavior of a random walker [26]. The random walker starts on an initial node (or a set of source nodes simultaneously), and moves to a neighboring node based on the probabilities of the connecting edges. The random walker may also choose to teleport to the start nodes with a certain probability, called the *restart probability*, *α*. The walking process is repeated at every time tick for a certain amount of time. At the end, the percentage of time spent on a node is proportional to its proximity to the starting nodes. The percentage of time spent is a probability distribution over the set of all nodes and changes in this distribution are modeled as a Markov chain. We refer to the stationary vector of the Markov chain as the *affinity vector*. The restart probability *α *enforces a restriction on how far the random walker moves away from the starting nodes. In other words, if *α *is close to 1, the local structure around starting nodes is analyzed, and as *α *gets close to 0, a more global view is observed. We use *α *= 0.7 for the results reported in this paper. A sketch of the random walk algorithm for finding the closest protein to a single protein is given in the Methods Section (Figure 1).

#### Repeated random walk algorithm

The random walk algorithm finds proteins that are in close proximity to a start node. Below we describe a linear combination technique to simulate a random walk starting from a set of proteins.

We can add the closest protein to the start set and repeat the random walk. Successive iterations can be used to identify clusters of any given size. Repeated random walks is based on this idea. However, the large number of random walks necessary to obtain a cluster in this way greatly reduces the speed of the algorithm. To lower the computational costs, the number of random walks performed can be reduced and the affinity vectors found using an alternative method.

Precomputed random walk results starting from single proteins in the set can be linearly combined to obtain the affinity vector for larger clusters starting from multiple proteins, as shown below.

**Theorem 1 ***Let ****P ****be the row normalized adjacency (transition) matrix defined by the graph, G*.

*Let ***s**_C _*be the restart vector for a set of nodes*, ***C***, *that contains a value of **in all entries corresponding to nodes in C, and 0 for other entries. Then, the stationary vector*, **x**_*C*_, *for a random walk with restarts starting from the set of nodes*, ***C***, *is *, *where ***x**_i _*is the stationary vector of random walk with restarts from node i*.

**Proof **The stationary vector **x**_i _of a random walk with restarts beginning from any single vertex, *i*, by definition, follows equation [28]:

(1)

Summing the above for all the nodes in C and dividing by *|C|*, we obtain,

(2)

Now, the stationary vector **x**_C _is defined to satisfy the equation,

(3)

Noting the form of Equations 2 and 3, and since the stationary vector is unique, we conclude that 

■

**Figure 2:**
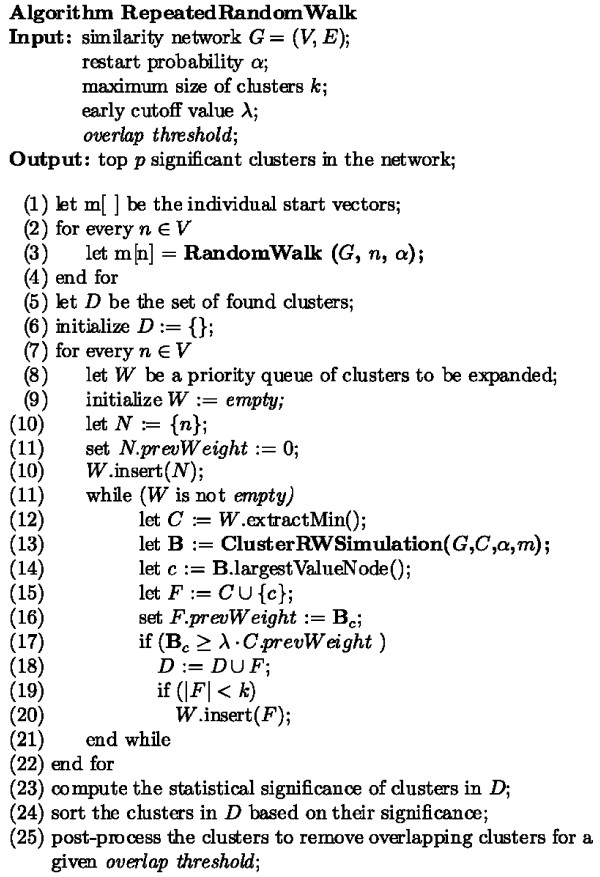
**Repeated random walk (RRW) algorithm**. Pseudocode for the overall RRW algorithm used to create significant clusters in the network.

**Figure 3:**
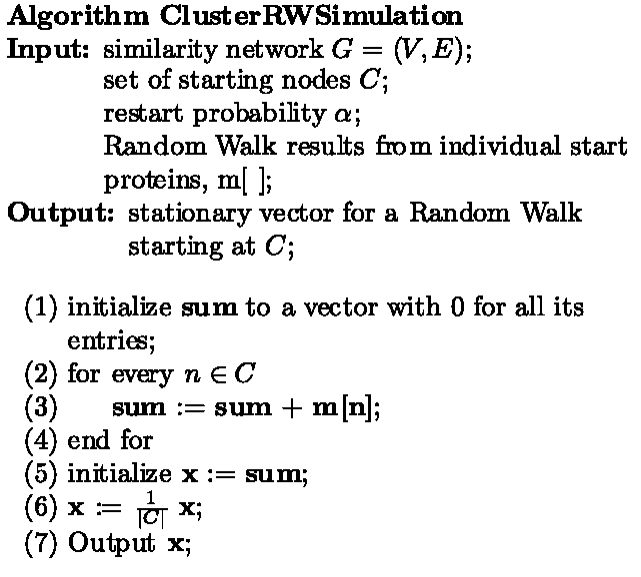
**Random walk from a cluster algorithm**. Pseudocode for the algorithm used to simulate a random walk with restarts from a cluster of vertices.

A sketch of the repeated random walk (RRW) and ClusterRWSimulation algorithms is given in the Methods Section (Figures 2 and 3). Starting from every node in the network, the *RandomWalk *method is run, and the resulting affinity vectors associated with each single node are saved. Sets of strongly connected proteins are then found by again starting from every node in the network and expanding the clusters repeatedly using the *ClusterRWSimulation *method. This method utilizes the vectors found in the *RandomWalk *method to quickly obtain the random walk affinity vectors, and the closest protein to the current cluster is found. This protein is added to the cluster, its score remembered, and resulting in a new cluster to be further expanded. This process is continued until either the next protein to be added's score is not within a given percentage, *λ *(the early cutoff), of the previously added protein's score, or we reach the maximum cluster size *k*. All clusters created during expansion are saved.

These expanded clusters are afterwards post-processed based on a given *overlap threshold*. The less significant of highly overlapping (redundant) clusters are then discarded. The overlap ratio between two clusters, *C*_1 _and *C*_2_, is given by *|C*_1 _∩ *C*_2_|/min {*|C*_1_|, |*C*_2_|} and is between 0.0 and 1.0.

The complexity of the Random Walk algorithm is linear in the size of the graph and maximum cluster size, *O *(|*V*|·*R *+ |*V*|·*k*), where *R *is the complexity of the RandomWalk algorithm, and the complexity of post-processing is *O*(*n*^2^) where *n *are the number of clusters created. The bottleneck for the RRW algorithm, in large graphs, are the calls to the RandomWalk method done in the beginning. On a protein network with *|V | *= 4,681 and *|E| *= 34,000, the random walk calls take about fifteen minutes in total (using a machine with a 3.2 GHz Intel Xeon CPU and 8 GB of RAM running the Ubuntu 8.04 operating system), versus less than a minute spent computing the clusters using the linear combination method after the Random Walk affinity vectors have been computed and stored.

In order to reduce this complexity, one can skip using the RandomWalk and simply use the best neighbors based solely on edge weights. However, this naïve nearest neighbor approach does not capture the structure of the network around starting nodes. Our experiments show that this is indeed the case.

### Statistical significance of a cluster

Given a set of proteins that form a cluster in a genome-scale protein network, we assign a statistical significance to that set. To create a quantitative representation of a cluster, we compute a score which is the average value of the random walk distance between all nodes in the cluster. (Since the affinity vectors from each node in the graph are already precomputed and stored during the RRW computations, this can be done quickly and efficiently.) Since the "distances" are the stationary probabilities, the average score value will range from 0 to 1.

The computation of significance of a score requires estimating the cdf of scores and computing p-value(s) = 1 - cdf(s). Score distributions can be computed empirically by sampling clusters of different sizes. However, we found that the typical scores we worked with had very small tail probabilities. For example, for a cluster size of 10, the mean was 3.27·10^-5^, the standard deviation was 1.28·10^-4^, and the tail probability had to be computed for a score of .0359, which is about 280 times the standard deviation removed from the mean. It is difficult to apply sampling to compute these small tail probabilities.

For our purposes, we assumed a simple relationship between the cdf, scores, and cluster sizes. Clearly, the cdf value of a score is monotonic in score. It is also monotonic in cluster size since the probability of a cluster having an average score less than a threshold increases with cluster size. We attempted a number of different estimates of cdf-values: (score·log *|C|*), (score·), and (score·|*C*|). Both (score·) and (score·|*C*|) were correlated significantly with biological significance in MIPS clusters (the percent of proteins in the cluster that belong to the same MIPS complex). For a sample size of 1,855 clusters, the Pearson Correlation Coefficient between the biological significance and (score·log *|C|*), (score·), and (score·|*C*|) was 0.00787, 0.158 and 0.229, respectively. Since the critical value of the correlation coefficient p for 1,855 items is 0.0763 at 0.001 probability, it can be seen that (score·) and (score·|*C*|) are both significantly correlated to biological significance.

In our experiments, a slower growing function of *|C| *(such as ) led to better precision and worse recall than a faster growing function of *|C| *(such as *|C|*). Choosing clusters with higher precision over recall, we adopted the  function and present results for p-value = 1 - (score·).

### Experimental results

In this section, we report our experimental results conducted on different variants of a *S. cerevisiae *protein interaction network, setting *λ *to be 0.6, *k *to be 11, and the overlap threshold to be 0.2. Varying the overlap threshold between 0.01 to 0.4 was found experimentally to affect the reported results only slightly, and so a value of 0.2 (one overlapping protein allowed in a cluster of size 5) was chosen. The values for *λ *and *k *were found to not significantly alter the majority of returned results as well, as the returned clusters tended to favor smaller sizes (on average 5-6 proteins). These values, however, were chosen after evaluating various parameter settings. For the model organism *S. cerevisiae*, we used the WI-PHI network by Kiemer *et al*. [29]. WI-PHI is a weighted undirected protein interaction network encompassing a large majority of yeast proteins. It is constructed by integration of various heterogeneous data sources such as application of tandem affinity purification coupled to MS (TAP-MS), large-scale yeast two-hybrid studies, and results of small-scale experiments stored in dedicated databases. The network contains 50,000 interactions for 5,955 yeast proteins. The weights, included in the original file, are determined by assessing each data source's performance in reproducing the results of a high confidence benchmark interactome. We also created noisy versions of these networks to demonstrate the robustness of RRW under noise.

#### Comparison to known MIPS complexes

In order to evaluate the performance of RRW, we use protein complexes from the MIPS complex catalog [27]. All proteins belonging to the same MIPS complex are determined to be interacting with each other. Two statistical results are obtained. First, the quality of a cluster is assessed by finding the percentage of proteins belonging to the same MIPS complex within that cluster. If multiple complex annotations are mapped to the same cluster, the annotation with the highest number of proteins contained in the cluster is chosen. In addition, benchmark protein complexes from the MIPS catalog were used to obtain precision, recall, and accuracy measures. The MIPS benchmark contains 49 protein complexes each of which contains 5 to 10 proteins. The goal was to find clusters as close as possible to the actual complex or pathway, as measured by: *precision = number of true positives/local cluster size*, *recall = number of true positives/size of complex or pathway*, and *accuracy *=  where true positives are proteins in the same benchmark complex which are found in the local cluster.

We compare our results to MCL [30] (using an inflation value of 2.5), as well as a naïve cluster expansion method we implemented. MCL has been identified as currently being the strongest graph clustering technique in two recent clustering survey papers [31,32], and so we focus our comparisons to this technique. The MCL inflation parameter was chosen after evaluating various parameter settings, as shown in Table 2. In the naïve expansion method, clusters are expanded by including the neighbors that are connected to the cluster with the largest weight edges. The main difference between this approach and the repeated random walk is that the naïve method chooses the closest neighbors based on local similarity, whereas RRW chooses the closest neighbors based on the global structure of the network.

**Table 2:** Precision, recall, & accuracy on pre-selected MIPS clusters with various MCL inflation parameter values

**Network**	**Precision**	**Recall**	**Accuracy**
	**2.0/2.5/3.0**	**2.0/2.5/3.0**	**2.0/2.5/3.0**
WI-PHI	0.471/0.512/0.524	0.858/0.832/0.780	0.636/0.657/0.639

FP40	0.469/0.538/0.605	0.859/0.768/0.837	0.635/0.643/0.711

FN40	0.400/0.423/0.432	0.719/0.670/0.628	0.537/0.532/0.521

Rewire40	0.455/0.480/0.550	0.666/0.565/0.461	0.550/0.520/0.504

Ignoring uncharacterized proteins and clusters less than 5 characterized proteins in size, 50% of all the reported clusters from RRW on the original network had at least 90% of their members from the same MIPS complex, significantly higher compared to the 17% in MCL or 7.8% in naïve, as can be seen from Table 3. In addition, three types of noisy networks were generated to observe the effect of false negatives (FN40), false positives (FP40), and edge shuffling (Rewire40) separately. FN40 network was obtained by randomly removing 40% of the edges in the original network. The FP40 network was obtained by adding 40% new random edges to the original network. And the Rewire40 network was obtained by shuffling 40% edges of the original network so that the degree distribution of the original network was preserved. Among these noisy networks as well, the quality of the majority of clusters remains significantly higher in RRW, proving its robustness against network noise, as seen from Tables 4, 5, and 6.

**Table 3:** Results for the WI-PHI network

**% in same MIPS category**	**RRW**	**MCL**	**Naïve**
90+%	50%	17%	7.8%

80+%	71%	30%	19%

70+%	72%	42%	31%

60+%	86%	57%	44%

50+%	91%	77%	70%

25+%	98%	99%	98%

**Table 4:** Results for the FP40 network

**% in same MIPS category**	**RRW**	**MCL**	**Naïve**
90+%	52%	20%	6.3%

80+%	72%	34%	17%

70+%	74%	50%	26%

60+%	84%	63%	37%

50+%	87%	82%	63%

25+%	99%	99%	96%

**Table 5:** Results for the FN40 network

**% in same MIPS category**	**RRW**	**MCL**	**Naïve**
90+%	47%	10%	7.7%

80+%	67%	23%	19%

70+%	68%	32%	26%

60+%	87%	49%	44%

50+%	91%	79%	63%

25+%	99%	98%	95%

**Table 6:** Results for the Rewire40 network

**% in same MIPS category**	**RRW**	**MCL**	**Naïve**
90+%	43%	17%	4.8%

80+%	64%	32%	13%

70+%	64%	46%	21%

60+%	77%	65%	33%

50+%	80%	81%	57%

25+%	97%	98%	93%

Table 7 shows the precision, recall, and accuracy values for the local clusters found in the *S. cerevisiae *network. The precision of RRW is again confirmed to be much higher than the other methods, emphasizing the quality of the clusters found. This precision in clustering is especially important in biological domains such as protein networks, as it enables more accurate predictions for proteins with unknown cellular function. The recall for RRW, however, is found to be low compared to both MCL and naïve. This means that, though the clusters found by RRW are highly precise, they may not find all proteins within a category, or may split single categories into multiple separate clusters. Comparing the average created cluster size of 5.72 for RRW, 9.82 for MCL, and 10.9 for the naïve method, it can indeed be seen that RRW created smaller clusters, leading to a lower recall rate. However, despite this, the overall accuracy measure of the RRW clusters are still higher than those found in both the MCL and naïve methods across all the networks.

**Table 7:** Precision, recall, and accuracy on pre-selected MIPS clusters

**Network**	**Precision**	**Recall**	**Accuracy**
	**RRW/MCL/Naïve**	**RRW/MCL/Naïve**	**RRW/MCL/Naïve**
WI-PHI	0.765/0.512/0.363	0.734/0.832/0.791	0.749/0.657/0.535

FP40	0.788/0.538/0.362	0.724/0.768/0.795	0.755/0.643/0.537

FN40	0.708/0.423/0.326	0.595/0.670/0.699	0.649/0.532/0.477

Rewire40	0.667/0.480/0.370	0.545/0.565/0.706	0.603/0.520/0.511

#### Comparison to known biological processes

In addition to MIPS complexes, proteins that are known to function in the same biological process were also used as a separate gold standard to further confirm that found clusters relate to biologically functional modules. A list of 295 significant GO biological process terms was used as given by Myers et al. [33] to specify the biological processes in a cell. We used the GO annotations [34] for yeast (May 23, 2009 version) to identify sets of proteins annotated with the same GO biological process term. 158 of the significant biological process terms were found to be annotated to at least 5 proteins. Hierarchical information was accounted for in this step by allowing proteins with an annotation lower in the tree to match with a parent annotation. These 158 terms, and the sets of proteins annotated with these terms, were then used as a gold standard. All proteins matching the same term were assumed to function together. Comparing the returned clusters in a manner similar to that used with the MIPS standard, it can be seen from Tables 8, 9, 10, and 11 that again the quality of clusters reported by RRW were significantly higher than most of those reported by MCL or the naïve method.

**Table 8:** Results for the WI-PHI network

**% same GO annotation**	**RRW**	**MCL**	**Naïve**
90+%	39%	6%	13%

80+%	60%	22%	26%

70+%	62%	32%	35%

60+%	76%	49%	57%

50+%	79%	67%	69%

25+%	96%	97%	98%

**Table 9:** Results for the FP40 network

**% same GO annotation**	**RRW**	**MCL**	**Naïve**
90+%	42%	18%	10%

80+%	60%	43%	21%

70+%	62%	58%	28%

60+%	76%	75%	46%

50+%	79%	85%	57%

25+%	95%	99%	95%

**Table 10:** Results for the FN40 network

**% same GO annotation**	**RRW**	**MCL**	**Naïve**
90+%	41%	4.5%	18%

80+%	60%	19%	32%

70+%	61%	31%	40%

60+%	81%	50%	61%

50+%	83%	68%	71%

25+%	97%	98%	99%

**Table 11:** Results for the Rewire40 network

**% same GO annotation**	**RRW**	**MCL**	**Naïve**
90+%	30%	13%	7.8%

80+%	49%	29%	21%

70+%	49%	35%	25%

60+%	64%	57%	40%

50+%	67%	70%	52%

25+%	92%	98%	91%

#### Analysis of select clusters for biological significance

To further validate the biological significance of the clusters discovered by RRW, we next discuss several statistically significant clusters discovered by our technique that are also biologically meaningful. One high scoring cluster found by RRW, and not created by either MCL or the naïve method, consisted of the proteins YML049c, YMR240c, YMR288w, YOR319w, and YPR094w. Though not all listed within the same MIPS complex, these 5 proteins were among the 7 found to interact in the yeast SF3b U2 snRNP subunits that associate with the pre-mRNA branchpoint region [35]. Another cluster found consisted of 5 proteins: YBL097w, YDR325w, YFR031c, YLR086w, and YLR272c. The MIPS complex catalogue did not list any of these five together in the same physical complex. However, their corresponding genes exactly match the 5 subunit *S. cerevisiae *condensin complex [36], essential for chromosome segregation during mitosis, demonstrating the ability of RRW to discover significant functional complexes as well as physical. Another 5 protein cluster discovered contained YDR200c, YFR008w, YLR238w, YMR029c, and YMR052w. Again, though not all contained within the same MIPS complex, these proteins have all been found to be part of a six-member group of interacting proteins that prevent recovery from pheremone arrest in yeast [37].

## Conclusion

In this paper, we proposed a novel algorithm based on repeated random walks on graphs for discovering functional modules within genome-scale protein networks. We applied the RRW on an interaction network of yeast genes by Kiemer *et al*. [29] and efficiently identified statistically significant clusters of proteins. We validated the biological significance of the results by comparison to known complexes in both the MIPS complex catalogue database [27] and GO functional annotations [34], as well as to existing clustering techniques. The repeated random walk technique offers significant improvements in precision over existing clustering techniques by making use of the strength of functional associations as well as the network topology and providing clusters of desired overlap ratio. Overlapping clusters proved a more accurate model of real biological networks with multifunctional proteins. In summary, our technique discovers biologically more significant clusters in a genome-wide protein interaction network using global connectivity and supporting evidence information accurately and efficiently.

## Methods

### The Random Walk and the Repeated Random Walk algorithms

Figure 1 gives the algorithm for finding the stationary vector of a Random Walk with restarts from a single starting node. The complexity of the algorithm is *O*(*w*·|*V*|^2^), where *w *is the number of iterations to converge. The value of *w *is determined by the structure of the network and the restart probability *α*. In general, the ratio of the first two eigenvalues of a transition matrix specifies the rate of convergence to the stationary probability [38].

The Repeated Random Walk (RRW) and Random Walk starting from a cluster (ClusterRWSimulation) algorithms are given in Figures 2 and 3. For the RRW algorithm, starting from every node in the network, sets of strongly connected proteins are found by expanding the clusters repeatedly using the ClusterRWSimulation method. Clusters of size ≤ *k *are inserted into a priority queue ordered by their statistical significance. For expanding a cluster *C*, the ClusterRWSimulation method is run and the closest protein in its stationary vector recorded. This neighbor protein is added to *C*, as long as its weight is within the early cutoff, *λ*, of the previously added protein to the cluster, resulting in one new cluster to be further expanded. The complexity is linear with the maximum cluster size, *O *(|*V*|·*k*).

*An implementation of the RRW algorithm is available for download at *

## Authors' contributions

KM implemented the Repeated Random Walk algorithm and performed the experiments. TC implemented the Random Walk algorithm (starting from a single node) and provided the datasets used in the experiments. AS worked on the underlying algorithms. All authors read and approved the document.
